# Bioinformatics Analysis of Global Diversity in Meningococcal Vaccine Antigens over the Past 10 Years: Vaccine Efficacy Prognosis

**DOI:** 10.3390/medsci11040076

**Published:** 2023-12-01

**Authors:** Viktoriia Yu. Savitskaya, Nina G. Dolinnaya, Vadim V. Strekalovskikh, Elizaveta S. Peskovatskova, Viktoriia G. Snyga, Vadim S. Trefilov, Mayya V. Monakhova, Elena A. Kubareva

**Affiliations:** 1 Department of Chemistry, Lomonosov Moscow State University, Leninskie Gory 1, Moscow 119991, Russia; 2 Department of Bioengineering and Bioinformatics, Lomonosov Moscow State University, Leninskie Gory 1, Moscow 119234, Russia; vadimstrek@yandex.ru (V.V.S.); elizavetapeskovatskova@gmail.com (E.S.P.); 3 Belozersky Institute of Physico-Chemical Biology, Lomonosov Moscow State University, Leninskie Gory 1, Moscow 119992, Russia

**Keywords:** *Neisseria meningitidis* serogroup B, meningococcal vaccine, bioinformatics tools, machine learning methods, multiple amino acid sequence alignments

## Abstract

*Neisseria meningitidis* (*N. meningitidis)* serogroup B (MenB) is the leading cause of invasive meningococcal disease worldwide. The pathogen has a wide range of virulence factors, which are potential vaccine components. Studying the genetic variability of antigens within a population, especially their long-term persistence, is necessary to develop new vaccines and predict the effectiveness of existing ones. The multicomponent 4CMenB vaccine (Bexsero), used since 2014, contains three major genome-derived recombinant proteins: factor H-binding protein (fHbp), *Neisserial* Heparin-Binding Antigen (NHBA) and *Neisserial* adhesin A (NadA). Here, we assessed the prevalence and sequence variations of these vaccine antigens in a panel of 5667 meningococcal isolates collected worldwide over the past 10 years and deposited in the PubMLST database. Using multiple amino acid sequence alignments and Random Forest Classifier machine learning methods, we estimated the potential strain coverage of fHbp and NHBA vaccine variants (51 and about 25%, respectively); the NadA antigen sequence was found in only 18% of MenB genomes analyzed, but cross-reactive variants were present in less than 1% of isolates. Based on our findings, we proposed various strategies to improve the 4CMenB vaccine and broaden the coverage of *N. meningitidis* strains.

## 1. Introduction

The bacterium *Neisseria meningitidis* (*N. meningitidis*) is an encapsulated, aerobic gram-negative diplococcus that can effectively attach to human mucosal cells and cause an acute infectious disease [1]. Transmission of the pathogen occurs by airborne droplets and leads to invasive meningococcal infection, manifested by meningitis and septicaemia [2]. However, most infected individuals experience a period of asymptomatic carriage of the bacterium in the upper respiratory tract. The disease is characterized by a relatively high mortality rate: 10–15% with antibacterial therapy and 50–80% without treatment [3]. In addition, almost 20% of surviving patients experience complications such as neurological disorders, hearing loss, paralysis or mental disorders [4]. Although incidence peaks in infants and adolescents one-third to one-half of cases occur in adults over 18 years of age [1].

To survive in the human body, *N. meningitidis* possesses a number of virulence factors. They help bacteria adapt to new conditions, avoid an immune response and acquire resistance to therapy [5]. One of the major virulence factors of *N. meningitidis* is their capsule polysaccharide, which determines the serogroup of the meningococcal strain [6]. Non-encapsulated strains rarely cause invasive disease and are found in asymptomatic carriers [7]. Capsular polysaccharide – glycoprotein conjugate vaccines have been shown to be effective against serogroups A, C, W and Y strains [8]. However, there is no vaccine-induced immunity against the serogroup B (MenB) strain, which causes more than a third of cases of the diseases because the serogroup B polysaccharide capsule mimics human-neural-cell adhesion molecules [9].

An alternative to capsular polysaccharide vaccines are vaccines targeting the outer membrane proteins of MenB strains. However, they have a significant limitation in the form of their highly effective antigenic variation [10]. For example, factor H-binding protein (fHbp) is required by *N. meningitidis* to evade the human immune response: it regulates the alternative complement pathway and protects the meningococci from complement-mediated phagocytosis and cell lysis [11]. This protein is one of the most suitable as the vaccine component because it is ready available, highly expressed, and capable of inducing bactericidal antibodies. The fHbp has been classified into three variants 1, 2 and 3, which are grouped into subfamily A (corresponding to variants 2 and 3) and subfamily B (corresponding to variant 1) based on sequence diversity [12,13]. All of them elicit protective-antibody responses in mice, rabbits, rhesus macaques and humans. However, antibodies produced against a particular fHbp variant generally do not cross react with other antigen variants, especially those from different subfamilies [14].

About a decade ago, the four-component vaccine 4CMenB (trade name Bexsero) containing recombinant fHbp from subfamily B was developed and licensed [15,16,17]. To form protection against *N. meningitidis* strains with fHbp from subfamily A, 4CMenB, in addition to fHbp, contains other genome-derived recombinant protein antigens: *Neisserial* adhesin A (NadA), *Neisserial* heparin-binding antigen (NHBA), as well as outer membrane vesicles preparation from the New Zealand strain [18]. This combination can provide wide strain coverage. However, outer membrane proteins have significant limitations due to high antigenic and phase variations in their genes, which reduces vaccine efficacy. Ongoing global surveillance is necessary to understand and predict dynamic changes in the epidemiology and biology basis of meningococcal disease and to make recommendations for current and future vaccines or other prevention strategies. Empirical assays of the binding efficiency of post-vaccination antibodies to *N. meningitidis* antigens are needed to identify cross-reactive vaccine candidates and increase strain coverage. However, in vitro and in vivo studies are too complex for global screening. Bioinformatic approaches to study antigen sequence diversity are suitable as a first step in identifying potential vaccine candidates. Currently, whole-genome sequencing of biomaterial from *N. meningitidis* clinical isolates has significantly expanded the databases in which sequences of bacterial antigens are presented [3].

In our study, we applied whole-genome sequencing data on variations in meningococcal vaccine antigens available worldwide over the past 10 years to evaluate the effectiveness of existing vaccines and identify new candidates to improve protection against meningococcal disease. We analyzed the sequence diversity of the three major components (fHbp, NadA and NHBA) of the four-component 4CMenB vaccine against MenB isolates whose genomes were obtained and deposited into the PubMLST database between 2013 and 2023. Using vaccine-reactivity information, multiple amino acid sequence alignments, and a Random Forest Classifier model as a machine learning method, we examined the variability of each antigen, assessed strain coverage for each antigen separately, and developed approaches to predict new and better vaccine options.

## 2. Materials and Methods

### 2.1. PubMLST Database Application for Searching N. meningitidis Isolates

To analyze the information about genetic materials of MenB isolated and collected in a database since 2013, the list of *N. meningitidis* isolates was obtained by searching pages Home > Organisms > *Neisseria* spp. > Genome collection > Search or browse database. Entered search criteria: “species” is *Neisseria meningitidis*, “year” from 2013 to 2023, “serogroup” B.

Using the analysis tool “Breakdown”, plugin “Fields” we analyzed country/disease or age distribution.

### 2.2. Analysis of 4CMenB (Bexsero) Efficiency against of N. meningitidis Isolates

Using the analysis tool “Breakdown”, plugin “Fields” and scheme “Bexsero_reactivity”, we analyzed the efficiency of the vaccine against *N. meningitidis*. The deduced vaccine antigen reactivity (MenDeVAR) index was developed in PubMLST to show the reactivity of an isolate or antigen. The protein variants found in the genome are determined and the vaccine reactivity results were given as MenDeVAR: “Exact match”—isolate contains ≥ 1 exact sequence match to antigenic variants found in the vaccine; “Cross-reactive”—isolate contains ≥1 antigenic variant deemed cross-reactive to vaccine variants through experimental studies; “None”—all the isolate’s antigenic variants have been deemed not cross-reactive to vaccine variants through experimental studies; “Insufficient data”—isolate contains antigens for which there is insufficient data or antigens which are yet to be tested in experimental studies. As a result, we created Table 1 using Bexsero reactivity to estimate common efficiency of the vaccine.

### 2.3. Long-Term 4CMenB Reactivity of N. meningitidis Isolates

To investigate how different responses of *N. meningitidis* isolates to the 4CMenB vaccine have changed over the past 10 years, we estimated the number of isolates with a positive/negative or unknown reaction to the 4CMenB using MenDeVAR in PubMLST database per each year. Using Python script (https://colab.research.google.com/drive/1OAq8juqvTZEGKuFZPOGOqlS199N791fO?usp=sharing#scrollTo=N4_UPJszFj8M, accessed on 30 September 2023), we tested the dependence of the values of the proportion of isolates from different reactivity groups by implementing autocorrelation analysis. To test the statistical significance of the result, a Durbin–Watson statistic method was used. To calculate the isolate’s proportion, data from the years 1976–2022 were recorded. This choice of time period is justified by the fact that the number of isolates recorded for the 1940s, 1950s, and 1960s differed greatly from more recent data.

### 2.4. Bar Plots of Vaccine Antigen Alleles Distribution in the Target Population of MenB

Bar plots (Figures 3, 6 and 8) showing the distribution of antigen alleles (variant ID’s) in the target population were obtained using the analysis tool “Breakdown”, plugin “Fields” and the loci parameter with the value of the corresponding locus (fHbp_peptide, NHBA_peptide or NadA_peptide) of the PubMLST.

### 2.5. Phylogenetic Analysis of Vaccine Antigen Alleles MenB

The global tree of fHbp alleles and the tree of the ”Insufficient data” group of fHbp were constructed using the NGPhylogeny service (https://ngphylogeny.fr, accessed on 30 September 2023) with the following workflow: multiple alignments of amino acids sequences produced using the MAFFT algorithm, tree construction using the Neighbor joining algorithm, and visualization in the iTOL service (https://itol.embl.de, accessed on 30 September 2023). The global tree does not use branch lengths for simplification. Both trees were rooted at the midpoint. The tree of the ”Insufficient data” group shows ID numbers of fHbp peptides whose sequences correspond to subfamily B which are marked in red (the branch of the fHbp vaccine variant ID 1 shown as control), and antigen variants from subfamily A are shown in blue (the branch of fHbp ID 19 which is a representative of the “None” group is shown as a control).

Multiple alignments of amino acid sequences produced by the MUSCLE algorithm were used to create phylogenetic trees of the NadA and NHBA antigens. The trees were constructed using the Maximum Likelihood algorithm.

### 2.6. 4CMenB Reactivity of Antigens of N. meningitidis Isolates

The characteristics of each antigens variant are present in PubMLST database. Using the analysis tool “Breakdown”, plugin “Combinations”, “id” as a provenance field and “bexsero notes” as secondary metadata, we obtained the list of ID variants with particular reactivity (“Cross-reactive”, “None” or “Insufficient data”).

### 2.7. Multiple Alignments of Amino Acid Sequences of Vaccine Antigens

To obtain the amino acid sequences (FASTA files) of the antigen variant, the *Neisseria* typing plugin of PubMLST database was used. The multiple sequence alignments of amino acid sequences of antigens were produced using the MAFFT algorithm using the Clustal Omega service (https://www.ebi.ac.uk/Tools/msa/clustalo/, accessed on 30 September 2023).

### 2.8. Machine Learning Classifier

All alleles of a particular antigen were grouped by reactivity on 4CMenB. To predict the reactivity of fHbp and NHBA antigen alleles from the “Insufficient data” group, we applied machine learning methods. The mainly used tools are pandas and scikit-learn libraries of Python (script is available using the link https://colab.research.google.com/drive/19bd5C6f1UIznpSP29jK822Z0aPbKbjiM#scrollTo=da95cb63-f7cd-48cc-bd81-953fcb0af68b, accessed on 30 September 2023). The MAFFT alignment of all antigen alleles for which reactivity on 4CMenB is known was used as training data; then, the alignment was converted into a data frame, where each cell corresponded to an amino acid residue (or gap) in the alignment. Conversion of amino acids into numerical features was completed using two types of encoding: one-hot encoding and label encoding. Two different types of coding were chosen to mutually compensate for some of their disadvantages, which may affect the prediction result. The Random Forest Classifier model was used for prediction; the test data frame was an alignment of alleles from the insufficient data group. The train:test ratio was 21:360 for fHbp and 18:346 for NHBA. Cross-validation using the LeaveOneOut method was carried out on the train data frame during hyperparameter optimization. Then, evaluation metrics (F1-score and confusion matrix) were obtained after evaluation classifiers were applied to test data to predict reactivity. The results of reactivity predictions are presented in an xlsx-table format separately for the two types of data encoding for comparison.

### 2.9. 3D Visualization of Polymorphic Sites

Crystal structure images of fHbp peptide ID 1 complexes with monoclonal antibodies Fab 1A12 and F4B3 were created using PyMol tools from the PDB database (Fab 1A12: 5O14, F4B3: 6XZW). The structure of fHbp peptide ID 15 and 19 was derived from the sequence of fHbp peptide ID 15 and 19 using the Alpha Fold 2 program.

## 3. Results

**Figure 1:**
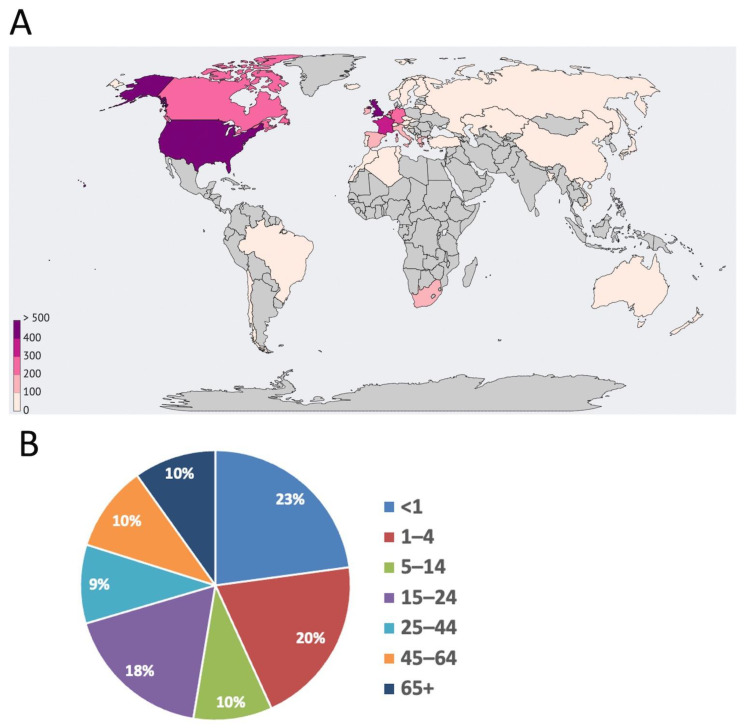
Distribution of MenB on the world map over the past 10 years (available via link https://pubmlst.org/bigsdb?db=pubmlst_neisseria_isolates&page=query&genomes=1, accessed on 30 September 2023) (**A**). Percentage of patients with meningitis and septicaemia as a function of age (**B**).

To date, more than 50 thousand genome sequences of *Neisseria* organisms have been deposited in the PubMLST database, more than half of which belong to *N. meningitidis*. PubMLST is the largest database that stores multi-loci sequence typing data, isolate information, and an ever-increasing number of complete genomic sequences for many microbes [19]. The subject of our study was the genomes of MenB isolates registered from 2013 to 2023, of which 5667 were found in the database (2 August 2023). Figure 1A shows the area of greatest distribution of MenB over the past 10 years, indicating the high prevalence of the pathogen in European countries, as well as in countries located in North America.

However, the “meningococcal belt” includes mainly African countries, where the risk of morbidity is extremely high, and epidemics of meningococcal infection occur every few decades [20]. The high level of development of the countries of Europe and North America makes it possible to accumulate and study a sufficient amount of biological material, while in African countries, where there is no universal vaccination coverage and antibiotic resistance is increasingly more common, such manipulations are difficult. As described above, most dangerous to human life is an invasive infection, leading to meningitis and septicaemia, which occur in no more than 15% of cases, according to the database. Severe disease is observed more often under the age of 24 years, especially often in infants (Figure 1B).

### 3.1. Response of N. meningitidis Isolates to the 4CMenB Vaccine

Today, vaccination is the most effective method of preventing meningococcal infections throughout the world. The four-component vaccine 4CMenB has become widely used since 2013. A scheme designed to monitor the target antigens included in the vaccine is based on comparing established peptide sequence variants with new ones. This scheme has provided information on the response (reactivity) of most *N. meningitidis* isolates to the 4CMenB vaccine [21]. The results are presented in Table 1.

According to the data obtained, the genomes of 33% of the studied isolates contain at least one antigen, the sequence of which exactly matches the sequence of the 4CMenB vaccine antigens, and 13% of the isolates contain at least one antigenic variant recognized as cross-reactive to the vaccine antigen. These data indicate that the 4CMenB vaccine is effective against at least 46% of *N. meningitidis* isolates. However, 10% of organisms in the population encode mutant forms of the antigens, and there is no vaccine-induced immunity against these forms of *N. meningitidis*. For the remaining 44% of isolates, there was insufficient information on vaccine reactivity.

**Figure 2:**
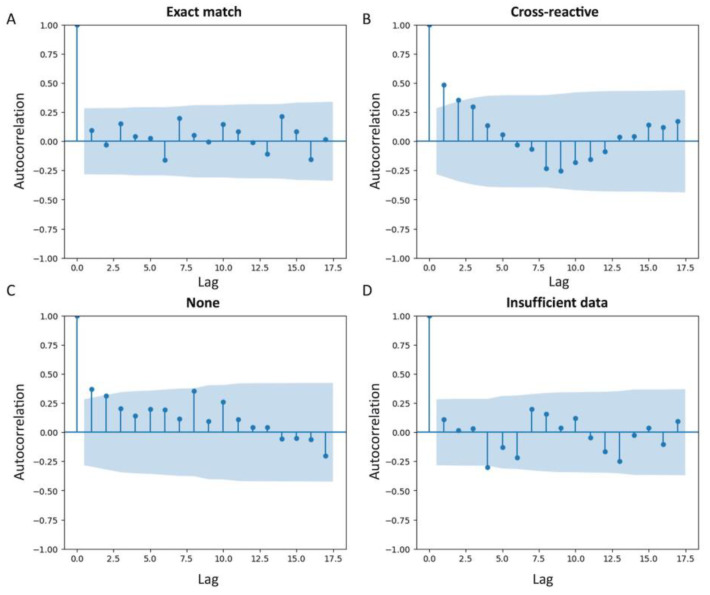
Autocorrelation plot of the changes in isolate reactivity to the vaccine over time. Proportion of isolates carrying vaccine antigen (“Exact match”) (**A**); proportion of isolates that were cross-reactive (**B**); proportion of isolates that are not cross-reactive (“None”) (**C**); the proportion of isolates for which vaccine reactivity information is not available (“Insufficient data”) (**D**).

We also assessed how the number of isolates with one or another type of reactivity to the 4CMenB vaccine has changed over the past 10 years (Figure 2). According to autocorrelation plots constructed for data from the years 1976 to 2022, there is no serial correlation for the values of the proportions of isolates in different years. The Durbin–Watson statistics for the “Cross-reactive”, “None”, “Exact match”, “Insufficient data” groups take the following values: 0.2297, 0.3788, 1.0607, 0.9335, respectively. This result may indicate the presence of weak positive autocorrelation in the “Cross-reactive” and “None” groups. However, the considered sample is too small, and the Durbin–Watson criterion may work incorrectly [23].

The 4CMenB vaccine consists of the variant antigens fHbp (ID 1), NadA (ID 2) and NHBA (ID 8). ID refers to a specific amino acid or nucleotide sequence of a protein or gene (allele), respectively. ID can be used to judge the conservation of a protein: the more resistant an antigen is to changes in the population, the fewer ID numbers will be assigned to its gene. To further explore vaccination coverage and predict potentially more effective antigen variants, we examined the diversity of each vaccine antigen separately.

### 3.2. Diversity and Distribution of fHbp

**Figure 3:**
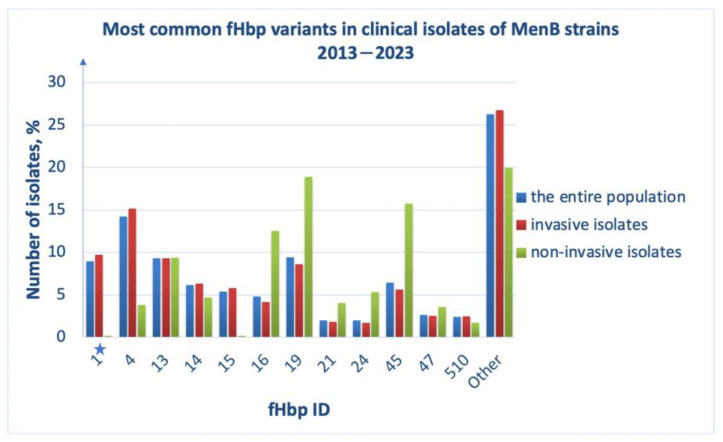
Most common fHbp protein variants in clinical isolates of MenB strains, 2013–2023, among the entire population (blue bars), among invasive isolates (red bars), and among non-invasive isolates (in patients with asymptomatic carriage, green bars); ID numbers of fHbp are indicated (*X*-axis). The asterisk indicates ID 1, the vaccine variant of fHbp. The proportion of isolates (*Y*-axis) was defined as the ratio of isolates with a certain fHbp ID to the total number of isolates reported in the analyzed group.

MenB isolates, 2013–2023, contain the fHbp genes encoding the protein variants that can be divided into two subfamilies (A and B) (File PDF S1), which is consistent with the previously obtained data [18]. Over the past 10 years, 383 different fHbp variants (alleles) have been identified in isolates deposited to the PubMLST database: fHbp ID 4 (subfamily B) is the most common, while the vaccine antigen variant fHbp ID 1 (subfamily B) is the fourth most common (Figure 3, blue bars). As for the isolates causing invasive meningococcal infections, the majority still express fHbp ID 4 but the fHbp variant ID 1 is already the second most common in this sample (Figure 3, red bars). The biological material of asymptomatic carriers of the infection contained bacteria whose genomes encoded predominantly fHbp ID 19 (subfamily A) and the vaccine variant of the antigen was not one of the most common alleles in this sample (Figure 3, green bars).

The most common fHbp variants shown in the plots in Figure 3. Subfamilies A and B include variants with IDs 45, 47, 19, 16, 21, 24 and 1, 4, 14, 15, 13, 510, respectively (File PDF S1). The PubMLST database contains information on only three isolates that have lost fHbp in their genome, confirming the high potential of the fHbp antigen as a vaccine component. 

Some fHbp protein variants have been experimentally proven to be cross-reactive with the vaccine antigen variant; their sequences are assigned ID numbers 15, 4, 14, 37, 144, 110, 215, 10, 12. It can be seen that three of them are included in the list of the most common fHbp protein variants in MenB strains, 2013–2023 (ID numbers 4, 14, 15 in Figure 3): 25% of isolates were identified whose genomes contained genes for one of these three fHbp antigen variants. We then compared the amino acid sequences of the cross-reactive fHbp proteins with the sequence of fHbp ID 1 used as a vaccine component (Figure S1). Both in the C-terminal region of the protein and in the N-terminal domain, a large zone with 100% conserved regions can be distinguished. Polymorphic sites in which the frequency of amino acid substitutions (a.a. substitutions) exceeds 30% are highlighted in green. Taking into account the cross reactivity of the analyzed fHbp variants, we can conclude that these polymorphic sites either do not affect the antibody–antigen interaction or they increase the efficiency of their binding. All cross-reactive fHbp variants belong to subfamily B, to which the vaccine variant, fHbp peptide (ID 1), also belongs (File PDF S1). Despite the high degree of conservation of all cross-reactive variants (more than 90%), one of them, fHbp peptide (ID 15), was slightly more varied from the vaccine variant (fHbp peptide ID 1) (File PDF S1). The remaining fHbp sequences differ from the fHbp peptide ID 1 sequence by less than 10%, although they may be less similar to each other. The a.a. substitutions described above do not appear to significantly affect fHbp conformation. Using the AlphaFold colab service, we created a model of the three-dimensional structure of the fHbp peptide ID 15 (File PDF S1). It did not differ significantly from the crystal structure of the fHbp peptide ID 1 obtained using X-ray diffraction analysis (PDB code 3KVD) [24].

From the PubMLST database, we extracted sequence information for fHbp variants that do not cross react with the vaccine antigen (group “None”). Their ID numbers are given in Table 2, which shows the percentage of fHbp genes in the genomes of the MenB population, 2013–2023.

**Figure 4:**
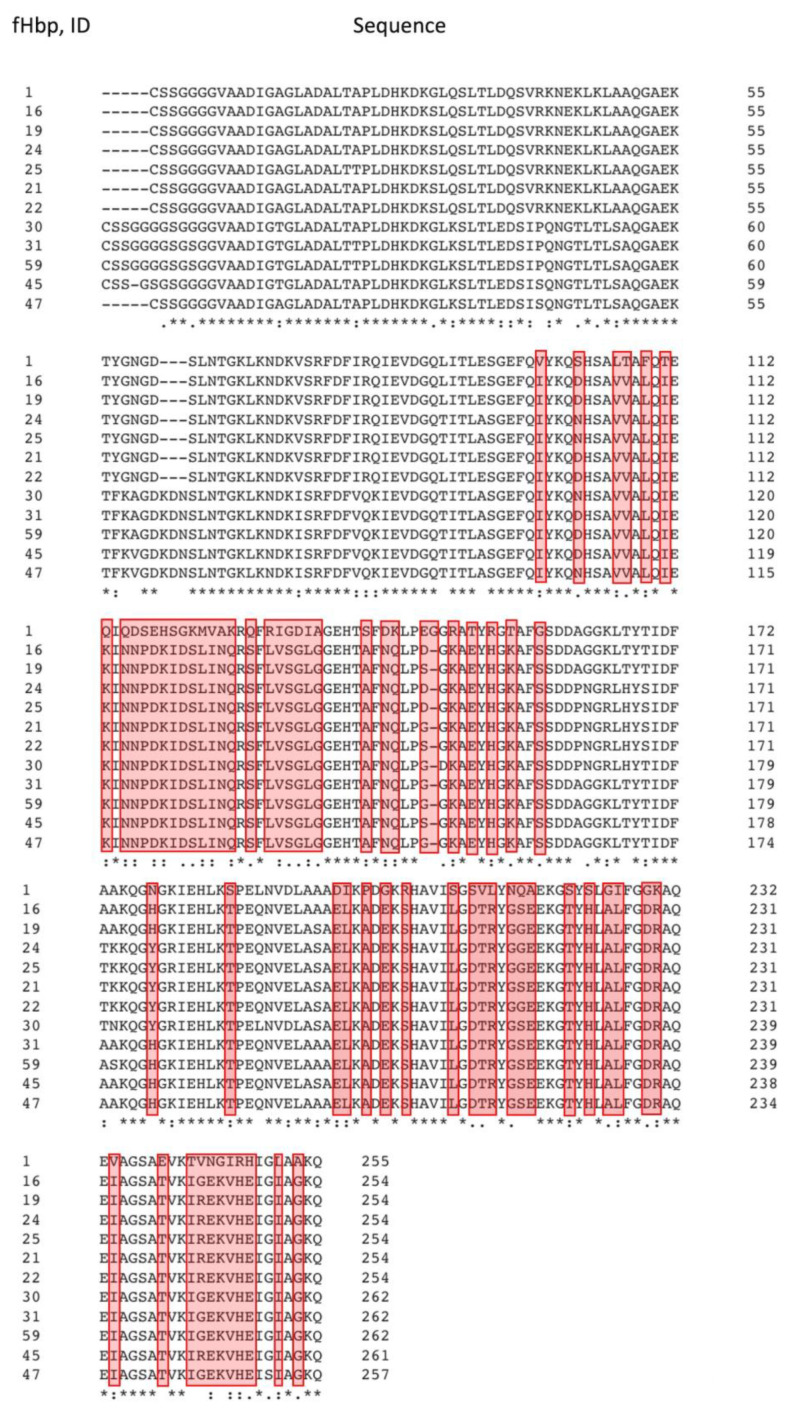
Alignment of amino acid sequences of non-cross-reactive fHbp variants from MenB strain isolates (2013–2023) and the fHbp peptide (ID 1) used in the 4CMenB vaccine. Clustal Omega service was applied for alignment (https://www.ebi.ac.uk/Tools/msa/clustalo/, accessed on 30 September 2023). An asterisk “*” indicates fully conserved amino acid position; a colon “:” indicates positions of amino acids with similar properties; dot “.” indicates positions of amino acids with weakly similar properties. Polymorphic sites found in 100% of cases are indicated in red (i.e., all fHbp protein variants belonging to the “None” group have a.a. substitutions at given positions compared to the fHbp peptide ID 1). The number of the last amino acid residue in the line is indicated on the right.

As expected, the fHbp members of the “None” group belong to the subfamily A and are quite distant from cross-reactive fHbp variants on the phylogenetic tree (File PDF S1). Six of these variants were revealed to be among the most common fHbp in the MenB population (IDs 19, 45, 16, 47, 24 and 21), and were also expressed by more than 45% of MenB isolates, 2013–2023, indicating a high risk of reduced vaccine effectiveness against *N. meningitidis*. We found a large number of polymorphic sites in the fHbp sequences in the C-terminal domain (amino acid residues S140-A253), as well as a variable zone in the N-terminal region (S100-G158). Figure 4 shows the a.a. substitutions found in all fHbp proteins belonging to the «None» group: it is these polymorphic sites that potentially form the mutant types of proteins that are poorly recognized by antibodies. The identity of the amino acid sequences of fHbp from the «None» group relative to that of the vaccine fHbp peptide ID 1 does not exceed 73%, although fHbp variants are identical to each other on average by 92%. 

With the exception of *N. meningitidis* fHbp variants, which belong to the “Cross-reactive” and “None” groups, information on antigen reactivity may not be available. In this case, fHbp variants fall into the group ”Insufficient data”. Compared to the other groups (“Cross-reactive” and “None”), the group named “Insufficient data” includes many more fHbp variants (more than 300 ID numbers), making up the largest group. This is easily explained: the database is constantly updated with information about the genomes of newly registered isolates, which happens much more often than conducting an experiment to determine the reactivity of a new fHbp variant. Among representatives of the ”Insufficient data” group in the MenB population, 2013–2023, fHbp variants IDs 13 and 510 are very common. We analyzed the amino acid sequences of fHbp proteins from the ”Insufficient data” group to predict their reactivity to the 4CMenB vaccine antigen. Based on the constructed phylogenetic tree, one can judge the distribution of fHbp variants of this group in subfamilies (File PDF S2). The ID numbers of fHbp peptides whose sequences correspond to subfamily B are marked in red (the branch of the fHbp vaccine variant ID 1 shown as control), and antigen variants from subfamily A are shown in blue (the branch of fHbp ID 19 which is a representative of the “None” group, is shown as a control). The fHbp variants IDs 13 and 510 belong to subfamily B, and, although they are quite far from the vaccine antigen on the phylogenetic tree, they are likely to be cross-reactive. Overall, the set of MenB fHbp variants in the “Insufficient data” group was divided almost equally between the two subfamilies. Previous data suggest that, in general, the subfamilies are not cross-reactive with each other: that is, antibodies produced after immunization with the 4CMenB vaccine, containing a subfamily B antigen will not provide effective protection against strains carrying a subfamily A antigen in the genome [22]. Using multiple amino acid sequence alignments of fHbp from all groups and machine learning algorithms (see Section 2 Materials and Methods), data on the potential reactivity of fHbp from the “Insufficient data” group were obtained. It turned out that about 49% of the analyzed antigen sequences were potentially not cross-reactive, and the remaining 51% were identified using artificial intelligence as cross-reactive (consistent results obtained using the two algorithms). The ID numbers of fHbp alleles and their predicted reactivity are available in File XLSX S1.

Taking into account the results obtained from the analysis of multiple fHbp amino acid sequence alignments, analysis of phylogenetic trees and the use of machine learning, it can be assumed that in approximately half of *N. meningitidis* isolates the fHbp variant is not cross-reactive. To create a wider coverage of pathogen strains with the 4CMenB vaccine, one could change its composition: (i) select an fHbp variant, after immunization with which the produced antibodies will recognize antigens of both subfamilies, and (ii) add an fHbp variant from subfamily A (or its C-terminal domain) to the vaccine. The information obtained about polymorphic sites (see Section 3.3) in the sequences of “None” group antigens, as well as their comparison with a.a. substitutions in the sequences of cross-reactive variants, may help in the future to solve the problem associated with vaccine resistance.

### 3.3. Analysis of Polymorphic Sites in Amino Acid Sequences of fHbp Proteins from the “Cross-Reactive” and “None” Groups

Table S1 shows polymorphic sites in the C-terminal region that occur at different frequencies in fHbp sequences from the “Cross-reactive” and “None” groups. A.a. substitutions occurring in 100% of the fHbp sequences of the “None” group may be responsible for reducing the efficiency of interaction with antibodies formed after immunization with 4CMenB. For example, the N215G substitution is described in the articles as reducing the affinity of fHbp to one of the antibodies [13]. This substitution is not found in any member of the “Cross-reactive” group. In addition, amino acids (for example, D142, R149, D197) in fHbp sequences from the “None” group have been described, whose substitutions are critical for antigen–antibody interaction [25,26]; similar polymorphic sites are also found in the sequences of cross-reactive fHbp variants, although with a reduced frequency. Substitutions E146D/S/G and V243R/G are found in all fHbp variants from the “None” group, while in representatives of the “Cross-reactive” group, substitutions E146K and V243A occur with a frequency of 67%. It is worth noting that among all fHbp variants studied, the 180-KIEHLK-185 motif is highly conserved; however, the K180R substitution was found in less than 50% of fHbp variant sequences from the “None” group. Such substitutions can affect the interaction of the antigen with the antibody; however, apparently, a single polymorphism does not lead to a loss of cross reactivity.

Despite the low conservation and lack of cross reactivity, the three-dimensional structure of fHbp peptide ID 19 (the most abundant among representatives of the “None” group), predicted using the AlphaFold colab service does not differ significantly from the crystal structure of the antigen, which is a component of the vaccine (fHbp peptide ID 1) (PDB file S1). Indeed, the C-terminal region, significantly altered in representatives of the “None” group, is responsible for the interaction of fHbp with the majority of antibodies induced after immunization with the 4CMenB vaccine.

**Figure 5:**
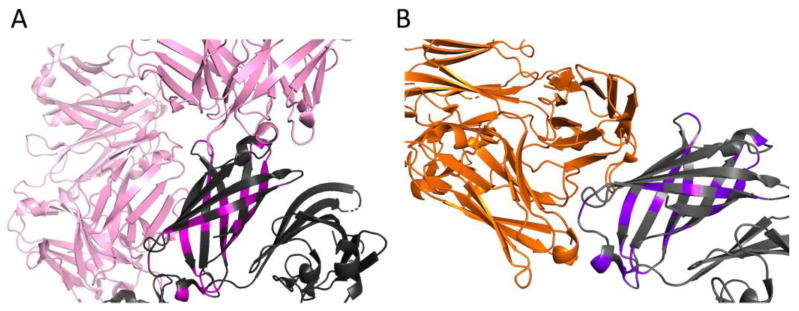
Crystal structure of the complex of fHbp peptide ID 1 (dark gray) with monoclonal antibodies induced after human immunization with the 4CMenB vaccine. (**A**) Fab 1A12; PDB: 5O14 [13] (pink) in complex with the fHbp vaccine variant (dark gray). The positions of the fHbp polymorphic sites indicated in magenta. (**B**) F4B3; PDB: 6XZW [25] (orange), complexed with the vaccine fHbp antigen (dark gray). The positions of polymorphic sites are shown in purple. Visualization of the structures was obtained in PyMol.

As an example of visualization of antigen–antibody interactions, Figure 5A shows a fragment of the crystal structure of the human monoclonal antibody Fab 1A12 (pink) in complex with the fHbp vaccine variant (dark gray), as well as the positions of the fHbp polymorphic sites (magenta) found in all variants from the “None” group (Table S1). Similarly, Figure 5B shows the structure of another antibody, F4B3 (orange), complexed with the vaccine fHbp antigen (dark gray), and the positions of the polymorphic sites are shown in purple. It can be seen that the sites of polymorphisms of fHbp peptide sequences from the “None” group are localized near the surface of the antigen that makes contact with the antibody.

For this reason, even without changing the overall structure of fHbp, variations in the amino acid sequence of the C-terminal region can locally influence the formation of antigen–antibody contact.

### 3.4. Prevalence and Diversity of NHBA

**Figure 6:**
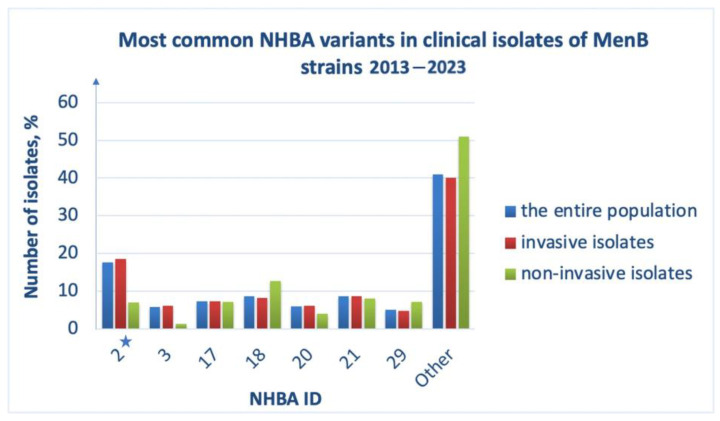
Most common NHBA protein variants in clinical isolates of MenB strains, 2013–2023, among the entire population (blue bars), among invasive isolates (red bars), and among non-invasive isolates (in patients with asymptomatic carriage) (green bars). ID numbers of NHBA are indicated (*X*-axis). The asterisk indicates ID 2-the vaccine variant of NHBA. The proportion of isolates (*Y*-axis) was defined as the ratio of isolates with a certain NHBA ID to the total number of isolates reported in the analyzed group.

The *Neisserial* heparin-binding antigen is a lipoprotein exposed on the cell surface of *N. meningitidis*. The 4CMenB vaccine contains NHBA ID 2 to provide broader meningococcal strain coverage [27,28]. Over the past 10 years, 366 different NHBA variants have been reported in the PubMLST database (File PDF S3). The most abundant is NHBA peptide ID 2 (Figure 6, blue bars). Isolates causing invasive meningococcal disease also express vaccine NHBA (Figure 6, red bars). In addition, NHBA variants IDs 21, 18, 17, 20, 3, 29 are common. MenB strains that do not cause invasive infection more often contain the gene of NHBA peptide ID 18 and the vaccine antigen was the fifth most common allele in this sample (Figure 6, green bars). Among the analyzed isolates, less than 0.1% of pathogens do not contain the NHBA gene in their genomes, which also confirms the advisability of its use as part of the 4CMenB vaccine. 

The PubMLST database contains information on experimentally proven cross reactivity of five variants of the antigenic NHBA peptides with IDs 10, 243, 1, 5 and 607. These alleles are not among the most common and occur no more often than in 5% of cases. Analysis of multiple alignments of the amino acid sequences of these variants demonstrates the high conservation of the C-terminal domain and the variability of the N-terminal part of NHBA antigens. Thus, the degree of identity of NHBA peptide IDs 2, 1 and 607, reaches 97–98%; although peptide ID 1 contains substitutions in the C-terminal domain: R367S, S373A, S399Y, G425D, G415S, K457R. Peptide ID 607 in addition to those indicated above, also has substitutions: S400T, R401K, P398S, A436V and Y472H. The NHBA peptide ID 5 was identical to the vaccine variant in 90% of the isolates, showing predominantly changes in the N-terminal domain (often in addition to the polymorphic sites described above). NHBA peptide IDs 10 and 243 differ to a greater extent from the other cross-reactive variants under consideration precisely in the N-terminal region, namely the absence of amino acid residues in the sequence range 110–175, which, however, does not prevent the formation of cross reactivity (Figure S2). It has been noted that antibodies recognizing the N-terminal region of NHBA have less affinity for the antigen and are less cross-reactive, apparently because the N-terminal domain itself is less conserved [29]. 

There were slightly more variants of the NHBA antigen without cross reactivity: peptide IDs 6, 9, 17, 18, 30, 31, 43, 47, 63, 112, 120 and 197. These include alleles that are widespread in the MenB population, such as peptide ID 18 (about 9%) and ID 17 (7.5%); the rest form up to 7%. As a result, approximately 23% of isolates carry an NHBA antigen sequence that does not cross react with 4CMenB, limiting vaccination coverage of meningococcal strains. In addition, half of the isolates containing NHBA of this group (mostly peptides with IDs 18 and 17) carried fHbp variants that were not cross-reactive (fHbp peptides with IDs 19 and 45) (File XLSX S2).

Comparison of the amino acid sequences of NHBA peptides from the “None” group with the vaccine variant showed a higher number of polymorphic sites in the C-terminal region compared to cross-reactive variants (Table 3, Figure S3). In general, the identity of NHBA variants from the “None” group compared vaccine antigen is from 80 to 90%. Figure S3 shows the amino acid alignment and polymorphic sites of the C-terminal domain; those that occur in the sequence of each antigen from the “None” group are indicated in red. There are only three amino acid substitutions in this domain: P360S, S373A, G425D. Interestingly, they occur quite frequently in the sequences of cross-reactive variants. Polymorphic sites that are less common in NHBA variants from the “None” group are indicated in blue in Figure S3 and are listed in Table 3. No single polymorphism appears to result in loss of cross reactivity of these antigens.

As for the N-terminal region of the NHBA antigen, it is quite variable both in cross-reactive variants and in those of the “None” group. There are conserved regions in the first 60 amino acid residues, large deletions in the region 110–175, and high variability in the sequence range 240–300.

**Figure 7:**
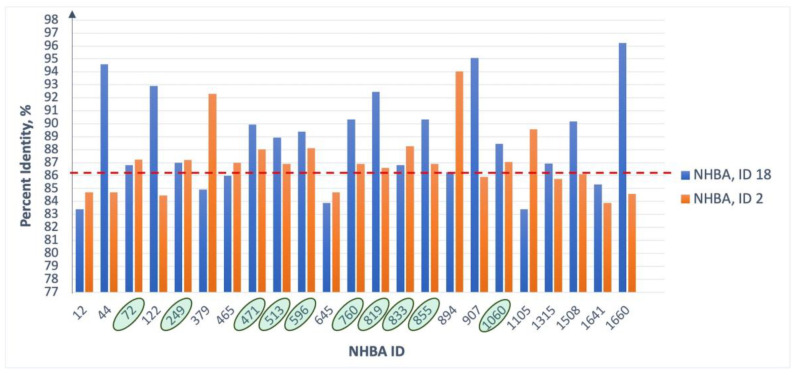
Percent sequence identity of NHBA variants found to be ambiguously reactive by machine learning compared to NHBA peptide ID 2 (vaccine variant, orange bars) and NHBA peptide ID 18 (most abundant member of the “None” group, blue) sequences. Green ovals show the ID numbers of those antigen variants whose amino acid sequences turned out to be quite close to the sequences of both the vaccine variant and the representative of the “None” group. The red dotted line shows the average degree of amino acid sequence identity of NHBA peptide ID 2 with the NHBA variants from the “None” group.

NHBA variants from the “Insufficient data” group are the most abundant compared to representatives of the “Cross-reactive” and “None” groups. They included NHBA peptides with IDs 20 and 21, which are among the most common antigen variants in MenB isolates, 2013–2023 (almost 15%) (Figure 6). Multiple amino acid sequence alignments of NHBA variants across all groups and machine learning algorithms were used to obtain potential reactivity data for the NHBA antigen from the “Insufficient data” group (File XLSX S3). Of the 346 studied representatives, cross reactivity was predicted for 75–80 antigens (depending on the encoding type used), and its absence was predicted for 266–271. Importantly, due to the large variability of NHBA, the level of reactivity of some variants has been ambiguously determined, but they may be of interest as new components of the vaccine. Cross-reactive NHBA variants from the “Insufficient data” group are on average 93% identical to the vaccine antigen. At the same time, the degree of identity of NHBA variants from the “None” group with the vaccine antigen is on average 86.2%, and with the most common representative of the same group, the NHBA peptide ID 18 is 89%. It can be assumed that those NHBA variants whose reactivity is ambiguously determined may be cross-reactive both with the NHBA peptide ID 2 (vaccine component) and with the common variant of the “None” group, for example, NHBA peptide ID 18. Figure 7 shows the comparative percentage identity of the amino acid sequences of NHBA variants, whose reactivity was ambiguously determined, and had the NHBA peptide IDs 2 and 18.

### 3.5. NadA Abundance and Diversity

**Figure 8:**
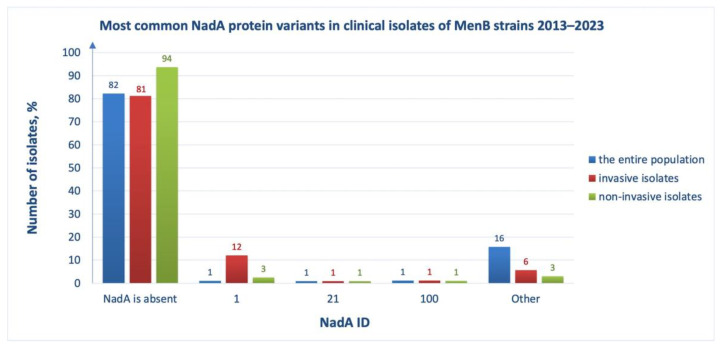
Most common NadA protein variants in clinical isolates of MenB strains, 2013–2023, among the entire population (blue bars), among invasive isolates (red bars), among non-invasive isolates (in patients with asymptomatic carriage) (green bars). ID numbers of NadA are indicated (*X*-axis). The vaccine variant of NadA is ID 8 (not one of the most common). The proportion of isolates (*Y*-axis) was defined as the ratio of isolates with a certain NadA ID to the total number of isolates reported in the analyzed group.

*Neisserial* adhesin A (NadA) is a homotrimeric protein that is involved in the process of adhesion and invasion into human epithelial cells during pathogenesis [26]. Only 18% of pathogenic meningococcal isolates has NadA (Figure 8, blue bars), but it is associated with invasive strains (Figure 8, compare red and green bars). Genomic analysis of isolates of meningococcal infection carriers shows that NadA was lost in more than 93% of cases (Figure 8, green bars).

**Figure 9:**
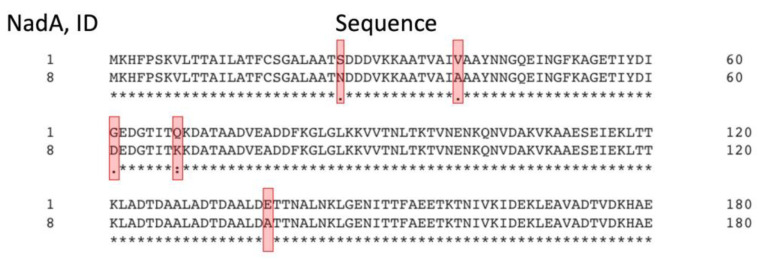
Comparison of the amino acid sequences of NadA the N-terminal domains from the NadA variants of ID 1 corresponding sequence of the NadA peptide ID 8 included in the 4CMenB vaccine. Red frames indicate the positions at which substitutions occur in the sequences of NadA variants with ID 1. Alignment of amino acid sequences constructed in Clustal Omega (https://www.ebi.ac.uk/Tools/msa/clustalo/, accessed on 30 September 2023). An asterisk “*” indicates fully conserved amino acid position; colon “:” indicates positions of amino acids with similar properties; dot “.” indicates positions of amino acids with weakly similar properties. The number of last amino acid residue in the line is indicated on the right.

It can be seen that the most common variant of NadA in isolates is ID 1, and the NadA vaccine antigen is assigned ID 8. Despite the narrow strain coverage by this component of the 4CMenB vaccine, the presence of NadA in its composition is reasonable because this protein is found predominantly in invasive strains that pose the greatest danger for a person. NadA was included in the 4CMenB vaccine as a homotrimeric protein lacking the C-terminal membrane anchoring domain [22]. Due to the small number of isolates carrying this antigen, only two cross-reactive NadA variants (IDs 3 and 6) and three variants from the “None” group (IDs 1, 21 and 100) were found in the database. Since NadA peptide ID 1 is the most abundant in the “None” group, we compared its amino acid sequence with that of the vaccine variant ID 8 (Figure 9) and found only five amino acid substitutions: N26S, A39V, D61G, K68Q and A138E in the N-terminal domain region included in the 4CMenB vaccine. Thus, cross reactivity of NadA antigens can only occur if the amino acid sequences of the N-terminal domains in the clinical isolates are extremely similar to those in the vaccine: even five sites of polymorphism lead to the absence of an immune response to this component of the vaccine (Figure S4).

We also examined multiple amino acid sequence alignments of the N-terminal domains of the “None” group members with each other and found that NadA ID 100 was completely identical to NadA ID 1, but the degree of identity of another variant (ID 21) was quite low (about 50%). Due to the lack of a representative sample of NadA isolates, it is difficult to predict the antigen sequence optimal for vaccine response. To increase the coverage of *N. meningitidis* strains, one could (i) replace the vaccine variant NadA ID 8 with the more common NadA ID1, or (ii) sacrifice NadA in favor of including the fHbp variant of subfamily A in the vaccine, since the coverage of strains with the first antigen will be, at best, 10–12%, and with the second antigen consisting of up to 49%.

## 4. Discussion

The multicomponent 4CMenB vaccine (Bexsero) has been used since 2014 to provide protection against the serogroup B meningococcal population over time, as vaccine targets tend to be quite conservative. 4CMenB contains three major genome-derived recombinant proteins: fHbp, NHBA and NadA that are capable of inducing bactericidal antibodies [30,31,32,33].

In our study, we evaluated the prevalence and sequence variability of these vaccine antigens in a panel of 5667 meningococcal isolates collected worldwide over the past 10 years and deposited in the PubMLST database. The protection induced by 4CMenB is known to depend on the degree of immunological cross reactivity between bacterial proteins and the corresponding vaccine antigens. We first assessed the change in isolate reactivity to the vaccine over time (for each year). The statistical analysis of time series performed using autocorrelation functions does not allow us to draw statistically significant conclusions about the existence of dependence between values at different time intervals. More data are needed for accurate analysis of time series and for predicting their behavior.

Among all vaccine antigens, fHbp turned out to be the most conserved in the isolates of the MenB population. The vaccine variant fHbp ID 1 (a representative of subfamily B) is one of the most abundant (Figure 3, blue and red bars). The amino acid sequences of cross-reactive fHbp antigens belonging to subfamily B are highly conserved (Figure S1). The detected polymorphic sites in the amino acid sequences of these fHbp variants do not appear to affect the antigen interaction with the antibodies induced after immunization with 4CMenB. On the other hand, all fHbp variants that do not demonstrate cross reactivity (“None” group) belong to subfamily A (File PDF S1). Some of them have become quite widespread in the MenB population over the last 10 years. The sequence of the C-terminal regions of fHbp variants from the “None” group of clinical isolates are significantly more variable (its identity was about 85%) compared to those of cross-reactive variants, the percentage of the identity of which reached 92%.

Polymorphic sites found in all fHbp variants that are not cross-reactive are shown in Table S1; they may contribute to the formation of a 4CMenB-resistant group of *N. meningitid* is isolates. The fHbp factor consists of two domains: a canonical C-terminal eight-stranded antiparallel β-barrel and an unusual “taco-shaped” N-terminal antiparallel β-sheet with high internal flexibility, connected through a 5-membered flexible chain linker [11]. The experimental results show that immunization with the 4CMenB vaccine produces a whole spectrum of monoclonal antibodies, but only a few of them potentially have cross reactivity to three evolutionary variants of fHbp. The RSCB PDB database contains crystal structures of *N. meningitidis* fHbp in complexes with several human monoclonal antibodies that exhibit cross reactivity (PDB codes: 6XZW, 5O14, 6H2Y). For example, Fab 1A12 (a monoclonal antibody obtained after human immunization with the 4CMenB vaccine) has been identified as an antibody capable of inducing complement-dependent elimination reactions of MenB strains carrying fHbp antigens. It is worth noting that the dissociation constant (*Kd*) of the Fab 1A12 complex with fHbp peptide ID 1 from subfamily B is almost 7–10 times lower than the *Kd* value for the antibody complex with representatives of subfamily A. The authors found that fHbp interaction with Fab 1A12 is mediated by the C-terminal β-barrel of fHbp, and single a.a. substitutions N215G, N190A and K185A reduce the affinity by 10, 50 and 30 times, respectively [13]. In our study we found that each member of “None” group have N215G, but no one can use this substitution in sequences of “Cross-reactive” group. Another human monoclonal antibody that also showed cross reactivity to several antigen variants, such as Fab 4B3, interacts effectively with fHbp, also through the C-terminal region of the antigen. The critical amino acid residues of fHbp for interaction with this antibody were E146, R149, K175, Q176, N178, and E218, single substitutions of which resulted in a tenfold increase in *Kd* value [25,26]. According to our study, these substitutions were more common in sequences of the “None” group compared to cross-reactive representatives. We claimed that this single polymorphism does not lead to a loss of cross reactivity. Comparison of the sequences of different *N. meningitidis* fHbp variants, the genes of which are contained in the genomes of MenB isolates, 2013–2023 (Table S1), as well as the available literature data [13,25,26], allow us to predict amino acid residues potentially involved in the formation of vaccine response.

Analysis of multiple amino acid sequence alignments of fHbp variants and the use of machine learning helped to conclude that subfamily A fHbp variants are not cross-reactive to the vaccine (File PDF S2, Table S1). In general, 4CMenB is effective against strains carrying the subfamily B antigens, which cover about half of the MenB isolates, 2013–2023. The results obtained support the need to improve the vaccine composition, which can be modified by replacing the fHbp peptide ID 1 with another variant from the subfamily B, having an amino acid sequence more similar to the sequence of the fHBp antigen belonging to the A subfamily. This strategy may facilitate broader pathogen coverage because multiple polymorphic sites from the “Cross-reactive” and “None” groups co-occur in fHbp peptide sequences. Another way to increase the effectiveness of the vaccine is to add an additional component to 4CMenB in the form of an antigen from subfamily A (or its C–terminal part, since antibodies mainly recognize this region of the antigen).

The four-component 4CMenB vaccine contains other *N. meningitidis* antigens in addition to the fHbp component to provide broader meningococcal strain coverage. *Neisserial* heparin-binding antigen is a lipoprotein exposed on the cell surface of *N. meningitidis*. NHBA has been shown to participate in biofilm formation as well as in adhesion to host epithelial cells [27]. Presumably, the structure of the N-terminal region of the antigen that binds heparin is disordered and rich in arginine, and the C-terminal domain has an antiparallel β-barrel fold. It has been proven that the vaccine version of NHBA peptide ID 2 induces bactericidal antibodies in humans that can interact with both the C- and N-termini of the antigen, inducing the formation of immune protection against meningococcal infection. This is of particular importance in relation to isolates with fHbp variants belonging to the subfamily A [28].

The amino acid sequence of NHBA is less conserved than fHbp mainly due to the N-terminal region, which is highly variable even in cross-reactive NHBA variants. The C-terminal region of this antigen, containing amino acid residues that bind to antibodies, is quite conserved in cross-reactive variants, but has many polymorphic sites in the isolates from the “None” group. Using a machine learning classifier, it was found that the available vaccine component (NHBA peptide ID 2) covers no more than 25% of *N. meningitidis* strains. Nevertheless, among the alleles there are NHBA variants whose reactivity is predicted ambiguously (File XLSX S3). The use of one of them (for example, ID 471, 596, 833) as a component of the vaccine makes it possible to increase the effectiveness of its action, since the described variants have a high degree of identity associated both with the vaccine NHBA ID 2 and with the non-cross-reactive NHBA peptide ID 18 (Figure 7).

*Neisserial* adhesin A (NadA) is an adhesive molecule which consists of a conserved C-terminal integral membrane β-barrel, which anchors proteins to the outer membrane, and an N-terminal domain responsible for adhesion and binding to host cellular receptors [26]. According to the literature of 2014, the antigen was present in approximately 30% of pathogenic meningococcal isolates, mainly in strains belonging to the hypervirulent MenB group. Today, the number of pathogenic meningococcal isolates has increased to 18% (Figure 8, blue bars), but they are still associated with invasive strains (Figure 8, red bars). NadA induces high levels of bactericidal antibodies in humans and is also recognized by serum antibodies from children [18]. The vaccine variant of the NadA peptide ID 8 is contained in less than 1% of pathogen isolates. In addition, cross-reactive NadA variants (ID 3 and 6) have also been extremely rare in the genomes of the MenB population, 2013–2023. This indicates extremely low coverage of *N. meningitidis* strains by this vaccine component. Due to the lack of a representative sample of antigens in isolates, a bioinformatics analysis of NadA sequences (including machine learning methods) and assessment of the necessity of this protein as a vaccine component is difficult. Based on our results of alignment of NadA alleles, it can be argued that the extremely low variability of its N-terminal domain is a decisive factor in the formation of antigen cross reactivity. NadA peptide ID 1 may provide the greatest coverage among all variants because it is the most abundant variant of the antigen and has a potential to be cross-reactive with NadA peptide ID 100 which is also widespread in the population. An alternative would be to exclude the NadA antigen from the vaccine to reduce the vaccine load in favor of including subfamily A fHBp.

In our study, we used multiple sequence alignment to predict variations in meningococcal vaccine antigens available worldwide over the past 10 years, and a Random Forest Classifier model as a machine learning method to predict strain coverage by 4CMenB. Using machine learning for evaluation of vaccine effectiveness has limitations compared to empirical assays. For example, this method does not take into account similarity of the chemical properties of substituted and substituting amino acid residues, which can significantly affect the antigen–antibody interaction. Besides this, our model has the train:test ratio of 21:360 and 18:346 for fHbp and NHBA, respectively, which is not enough for a good test size. The described points may be the source of errors. On the other hand, using a machine learning method to predict 4CMenB strain coverage has advantages: (i) it is not so complicated as in vitro and in vivo studies; (ii) it is suitable for the first step of global screening to find new and better cross-reactive vaccine variants; (iii) the prediction can be improved after accumulating additional results from empirical assay. The machine learning model’s prediction results may be useful in future research directions aimed at improving a vaccine against meningococcal disease, for example an in vitro analysis of antigen binding to antibodies elicited after immunization with a vaccine containing NHBA variants that were predicted using the machine learning model to be more cross-reactive (Figure 7). In addition, it is advisable to study ex vivo coverage of strains with a vaccine that, in addition to NHBA and fHbp, contains the most common variant of the *Neisserial* adhesin NadA ID 1 instead of NadA ID 8.

## 5. Conclusions

One of the widely used vaccines for the prevention of meningococcal disease is the multicomponent vaccine 4CMenB (Bexsero), which contains three antigens as its main components: fHbp, NHBA and NadA. We assessed the prevalence and sequence variations of these antigens in 5667 MenB isolates collected worldwide over the past 10 years and deposited in the PubMLST database. According to our data, 51% of the isolates were covered by the fHbp antigen and 25% by the NHBA antigen. The NadA antigen sequence was found in only 18% of MenB genomes, but cross-reactive variants were present in less than 1% of isolates. We described various strategies for each antigen studied using multiple amino acid sequence alignment analysis, bioinformatics tools, and a machine learning approach. We have demonstrated that the use of fHbp from two subfamilies or adhesin NadA ID 1 instead of NadA ID 8 could be the ways to improve the composition of the vaccine. Since the bioinformatics approach has some limitations, experimental verification of these predictions in the future is needed.

## Figures and Tables

**Table 1:** Percentage of MenB isolates that differ in their type of reactivity to the 4CMenB vaccine.

4CMenB Vaccine Reactivity	Exact Match	Cross-Reactive	None	Insufficient Data
Percentage, %	33	13	10	44

The designation “Exact match” means that the isolate has an antigen sequence that exactly matches the vaccine antigen sequence. “Cross-reactive” means that the isolate contains an antigen that has been found in experimental studies to cross react with vaccine variants (i.e., antibodies generated after immunization with 4CMenB effectively recognize the isolate antigen, causing an immune response). “None” means that all isolate antigens did not cross react with the vaccine variants (i.e., antibodies generated after 4CMenB immunization do not recognize the isolate antigen). “Insufficient data” means that the isolate contains antigens that have not yet been tested for reactivity to 4CMenB in experimental studies [22].

**Table 2:** Distribution of fHbp genes in the genomes of the MenB population, 2013–2023, and the products of which do not cross react with antigenic variants of the 4CMenB vaccine.

fHbp Peptide, ID	19	45	16	47	24	21	31	25	22	30	59
% of fHbp genes	9.48	6.47	4.84	2.63	2.03	1.97	1.29	1.00	0.48	0.30	0.12

**Table 3:** Polymorphic sites found in the amino acid sequences of NHBA variants from the “None” group; the NHBA vaccine antigen was used as a control.

**A.a. substitution**	* K457R	* G451S	**T445A/N**	**D438N**	* A436V	**T430K**	**D410N**	* R401K/G	* S400F/T
Frequency, %	86	15	15/15	7.5	30	25	7.5	23/23	23/46
**A.a. substitution**	* P397S	* S398Y	**T392M**	**T381A**	**E371K**	* R367S	**S339P**	**H338Y**	**G337E**
Frequency, %	46	54	7.5	7.5	7.5	46	7.5	7.5	7.5
**A.a. substitution**	**S334I**	**A332V**	**V328I**	**A323V**	**M315I**	**S307V**	**A305R**	**R302T/A**	**F301V/S**
Frequency, %	15	7.5	7.5	7.5	15	7.5	7.5	15/7.5	30/7.5

* Polymorphic sites that are also present in cross-reactive NHBA variants.

## Data Availability

Data associated with this manuscript are available in the Results, Materials and Methods and in the Supplementary Materials sections. Please contact the corresponding author to access the additional unpublished data.

## Supplementary Materials

The following supporting information can be downloaded at: https://www.mdpi.com/article/10.3390/medsci11040076/s1. Figure S1: alignment of amino acid sequences of cross-reactive variants of fHbp from isolates of MenB strains, 2013–2023, and fHbp peptide ID 1 used in the 4CMenB vaccine (the sequence is marked with a green dot). Alignment was completed in Clustal Omega service (https://www.ebi.ac.uk/Tools/msa/clustalo/, accessed on 30 September 2023). Green color indicates polymorphic sites that occur more often than in 30% of the sequences (i.e., at least 30% of the studied sequences of cross-reactive fHbp variants contain an amino acid substitution in this position). An asterisk “*” indicates fully conserved amino acid position, two dots “:” indicate positions with conservation between amino acids with similar properties and one dot “.” indicates positions with conservation between amino acids with weakly similar properties. The number of last amino acid residue in line is indicated on right. Table S1: polymorphic sites in the C-terminal region that occur at different frequencies in fHbp sequences from the “Cross-reactive” and “None” groups. Figure S2: comparison of amino acid sequences of cross-reactive NHBA variants (full-length) with the sequence of NHBA peptide ID 2 included in the 4CMenB vaccine. Polymorphic sites found in the C-terminal domain are indicated in green. Alignment of amino acid sequences constructed in Clustal Omega (https://www.ebi.ac.uk/Tools/msa/clustalo/, accessed on 30 September 2023). An asterisk “*” indicates fully conserved amino acid position, two dots “:” indicate positions with conservation between amino acids with similar properties, and one dot “.” indicates positions with conservation between amino acids with weakly similar properties. The number of last amino acid residue in line is indicated on the right. Figure S3: comparison of the amino acid sequences of NHBA C-terminal domains from the “None” group with corresponding sequence of the NHBA peptide ID 2 included in the 4CMenB vaccine. Red indicates the positions at which substitutions occur in the sequences of each NHBA of this group, and blue indicates the remaining polymorphic sites in the C-terminal domain. Alignment of amino acid sequences constructed in Clustal Omega (https://www.ebi.ac.uk/Tools/msa/clustalo/, accessed on 30 September 2023). An asterisk “*” indicates fully conserved amino acid position, two dots “:” indicate positions with conservation between amino acids with similar properties, and one dot “.” indicates positions with conservation between amino acids with weakly similar properties. The number of last amino acid residue in line is indicated on the right. Figure S4: the tree of the NadA. Multiple alignments of amino acids sequences produced using the MUSCLE algorithm to construct phylogenetic trees of the NadA antigen. The trees were constructed using the Maximum Likelihood algorithm and visualized in MEGA software. File PDF S1: the global tree of fHbp alleles. File PDF S2: the tree of the ”Insufficient data” group of fHbp peptides shows ID numbers of fHbp peptides whose sequences correspond to subfamily B are marked in red (the branch of the fHbp vaccine variant ID 1 shown as control), and antigen variants from subfamily A are shown in blue (the branch of fHbp ID 19 which is a representative of the “None” group, is shown as a control). File PDF S3: the tree of the NHBA. Multiple alignments of amino acids sequences produced by the MUSCLE algorithm to construct phylogenetic trees of the NHBA antigen. The trees were constructed using the Maximum Likelihood algorithm and visualized in MEGA software. PDB file S1: crystal structures of different variants fHbp—ID 1 (vaccine variant) ID 15 (cross-reactive) and ID 19 (“None” group). File XLSX S1: the prediction of 4CMenB reactivity of fHbp variants from “Insufficient data” group. File XLSX S2: combination of fHbp and NHBA variants in isolates genomes. File XLSX S3: the prediction of 4CMenB reactivity of NHBA variants from “Insufficient data” group.
