# Supplementary material for: Bioinformatics Analysis of Global Diversity in Meningococcal Vaccine Antigens over the Past 10 Years: Vaccine Efficacy Prognosis
Source: Med Sci (Basel). 2023 Dec 1;11(4):76. doi: 10.3390/medsci11040076 (PMC10744425; doi:10.3390/medsci11040076)
Supplement: Supplementary file 1 [file medsci-11-00076-s001.zip › Supplementary materials.pdf]

Supplementary materials

```
fHbp, ID                       Sequence

15        CSSGGGGSGGGGVAADIGAGLADALTAPLDHKDKGLKSLTLEDSISQNGTLTLSAQGAER    60
14        -----CSSGGGGVAADIGAGLADALTAPLDHKDKSLQSLTLDQSVRKNEKLKLAAQGAEK    55
10        -----CSSGGGGVAADIGAGLADALTAPLDHKDKGLQSLMLDQSVRKNEKLKLAAQGAEK    55
12        -----CSSGGGGVAADIGAGLADALTAPLDHKDKSLQSLTLDQSVRKNEKLKLAAQGAEK    55
215       -----CSSGGGGVAADIGAGLADALTAPLDHKDKGLQSLTLDQSVRKKEKLKLAAQGAEK    55
37        -----CSSGGGGVAADIGAGLADALTAPLDHKDKGLQSLMLDQSVRKNEKLKLAAQGAEK    55
4         -----CSSGGGGVAADIGAGLADALTAPLDHKDKSLQSLTLDQSVRKNEKLKLAAQGAEK    55
110       -----CSSGGGGVAADIGAGLADALTAPLDHKDKGLQSLTLDQSVRKNEKLKLAAQGAEK    55
1         -----CSSGGGGVAADIGAGLADALTAPLDHKDKGLQSLTLDQSVRKNEKLKLAAQGAEK    55
144       -----CSSGGGGVAADIGAGLADALTAPLDHKDKSLQSLTLDQSVRKNEKLKLAAQGAEK    55
               .****************************.*:** *::*: :: .*.*:*****:

15        TFKAGDKDNSLNTGKLKNDKISRFDFIRQIEVDGQLITLESGEFQVYKQSHSALTALQTE    120
14        TYGNGD---SLNTGKLKNDKVSRFDFIRQIEVDGQLITLESGEFQVYKQSHSALTALQTE    112
10        TYGNGD---SLNTGKLKNDKVSRFDFIRQIEVDGKLITLESGEFQVYKQSHSALTALQTE    112
12        TYGNGD---SLNTGKLKNDKVSRFDFIRQIEVDGQLITLESGEFQVYKQSHSALTALQTE    112
215       TYGNGD---SLNTGKLKNDKVSRFDFIRQIEVDGQLITLESGEFQVYKQSYSALTALQTE    112
37        TYGNGD---SLNTGKLKNDKISRFDFIRQIEVDGQLITLESGEFQVYKQSHSALTALQTE    112
4         TYGNGD---SLNTGKLKNDKVSRFDFIRQIEVDGQLITLESGEFQVYKQSHSALTALQTE    112
110       TYGNGD---SLNTGKLKNDKVSRFDFIRQIEVDGQLITLESGEFQVYKQSHSALTAFQTE    112
1         TYGNGD---SLNTGKLKNDKVSRFDFIRQIEVDGQLITLESGEFQVYKQSHSALTAFQTE    112
144       TYGNGD---SLNTGKLKNDKVSRFDFIRQIEVDGQLITLESGEFQVYKQSHSALTAFQTE    112
          *:  **   ***********:*************:***************:*****:***

15        QVQDSEHSGKMVAKRQFRIGDIVGEHTSFGKLPKDVMATYRGTAFGSDDAGGKLTYTIDF    180
14        QEQDPEHSGKMVAKRRFKIGDIAGEHTSFDKLPKDVMATYRGTAFGSDDAGGKLTYTIDF    172
10        QVQDSEDSGKMVAKRQFRIGDIAGEHTSFDKLPKGGSATYRGTAFGSDDAGGKLTYTIDF    172
12        QVQDSEDSGKMVAKRQFRIGDIAGEHTSFDKLPKGGSATYRGTAFGSDDAGGKLTYTIDF    172
215       QEQDSEHSGKMVAKRRFRIGDIAGEHTSFDKLPKGSSATYRGTAFGSDDAGGKLTYTIDF    172
37        QVQDSEDSRKMVAKRQFRIGDIAGEHTSFDKLPESDRATYRGTAFSSDDAGGKLTYTIDF    172
4         QVQDSEHSGKMVAKRQFRIGDIAGEHTSFDKLPEGGRATYRGTAFGSDDASGKLTYTIDF    172
110       QIQDSEHSGKMVAKRRFRIGDIAGEHTSFDKLPEGGRATYRGTAFGSDDAGGKLTYTIDF    172
1         QIQDSEHSGKMVAKRQFRIGDIAGEHTSFDKLPEGGRATYRGTAFGSDDAGGKLTYTIDF    172
144       QIQDSEHSGKMVAKRQFRIGDIAGEHTSFDKLPEGGRATYRGTAFGSDDAGGKLTYTIDF    172
          * ** *.* ******:*:****.******.***:.   ********.****.*********

15        AAKQGHGKIEHLKSPELNVDLAAADIKPDEKHHAVISGSVLYNQAEKGSYSLGIFGGQAQ    240
14        AAKQGHGKIEHLKSPELNVELATAYIKPDEKHHAVISGSVLYNQDEKGSYSLGIFGGQAQ    232
10        AAKQGHGKIEHLKSPELNVELATAYIKPDEKHHAVISGSVLYNQDEKGSYSLGIFGGQAQ    232
12        AAKQGHGKIEHLKSPELNVELATAYIKPDEKRHAVISGSVLYNQDEKGSYSLGIFGGQAQ    232
215       AAKQGYGKIEHLKSPELNVDLAAANIEQDEKHHAVISGSVLYNQDEKGSYSLGIFGEKAQ    232
37        AAKQGHGKIEHLKSPELNVDLAAADIKPDEKRHAVISGSVLYNQDEKGSYSLGIFGGQAQ    232
4         AAKQGHGKIEHLKSPELNVDLAASDIKPDKKRHAVISGSVLYNQAEKGSYSLGIFGGQAQ    232
110       AAKQGHGKIEHLKSPELNVDLAAADIKPDEKHHAVISGSVLYNQDEKGSYSLGIFGGKAQ    232
1         AAKQGNGKIEHLKSPELNVDLAAADIKPDGKRHAVISGSVLYNQAEKGSYSLGIFGGKAQ    232
144       AAKQGNGKIEHLKSPELNVDLAAADIKPDGKRHAVISGSVLYNQAEKGSYSLGIFGGKAQ    232
          ***** *************:**:: *: * *:************ *********** :**

15        EVAGSAEVETANGIRHIGLAAKQ---       263
14        EVAGSAEVETANGIHHIGLAAKQ---       255
10        EVAGSAEVETANGIHHIGLAAKQ---       255
12        EVAGSAEVETANGIHHIGLAAKQ---       255
215       EVAGSAEVKTANGIRHIGLAAKQ---       255
37        EVAGSAEVKTVNGIRHIGLAAKQEPL       258
4         EVAGSAEVETANGIRHIGLAAKQ---       255
110       EVAGSAEVKTVNGIRHIGLAAKQ---       255
1         EVAGSAEVKTVNGIRHIGLAAKQ---       255
144       EVAGSAEVKTVNGIRHIGLAAKQ---       255
          ********:*.***:********
```

Figure S1. Alignment of amino acid sequences of cross-reactive variants of fHbp from isolates of MenB strains, 2013-2023, and fHbp peptide, ID 1, used in the 4CMenB vaccine (the sequence is marked with a green dot). Alignment was done in Clustal Omega service (https://www.kelleybioinfo.org/algorithms/tutorial/TAli2.pdf). Green color indicates polymorphic sites that occur more often than 30% of the sequences (i.e., at least 30% of the studied sequences of cross-reactive fHbp variants contain an amino acid substitution in this position). An asterisk "*" indicates fully conserved amino acid position, two dots ":" indicate positions with conservation between amino acids with similar properties and one dot "." indicates positions with conservation between amino acids with weakly similar properties. The number of last amino acid residue in line is indicated on right.

Table S1. Polymorphic sites in the C-terminal region that occur at different frequencies in fHbp sequences from the "Cross-reactive" and "None" groups*.

**Frequency of substitution, %**	**Cross-reactive fHbp**	**None fHbp**
100	-	S140A<br>D142N<br>K143Q<br>G147-<br>**R149K**<br>T151E<br>R153H<br>G158S<br>S186T<br>D197N<br>T198L<br>P200A<br>G202E<br>R204S<br>S209L<br>S211D<br>V212T<br>I213R<br>**N215G**<br>G229D<br>K230R<br>G225A<br>I226L<br>S221T<br>S223H<br>T155K<br>I246V<br>R247H<br>H248E<br>L251I<br>T242I<br>N244E<br>G245K<br>A253G<br>V234I<br>E239T
50-99	**E146K**<br>**N178H**<br>G202E<br>R204H<br>A217D<br>K230Q<br>K241E<br>**V243A**<br>R247H	A162P<br>G163N<br>K165R<br>T167H<br>T169S<br>**N178H**<br>L189Q<br>D192E<br>A195S<br>Q217S<br>**V243R**
<50	D142G<br>G147D<br>G148V<br>**R149S**<br>G158S<br>**N178Y**<br>D192E<br>D197Y<br>D197N<br>R200Q<br>G229E	**E146D**<br>**E146S**<br>**E146G**<br>G147D<br>A174K<br>A174N<br>A174S<br>**N178Y**<br>**K180R**<br>Q217G<br>**V243G**

*Underlined polymorphisms are common for sequences of fHbp from "Cross-reactive" and "None" groups. Polymorphisms in red were mentioned in literature earlier as potentially important for antigen-antibody interaction [13, 25, 26]. The fHbp vaccine antigen (ID 1) was used as a control.

```
NHBA, ID                          Sequence

10          MFKRSVIAMACIFALSACGGGGGGSPDVKSADTPSKPAAPVVAEKETDAKEDAPQAGSQG     60
243         MFKRSVIAMACIVALSACGGGGGGSPDVKSADTLSKPAAPVVAEKETEVKEDAPQAGSQG     60
5           MFKRSVIAMACIFALSACGGGGGGSPDVKSADTLSKPAAPVVSEKETEAKEDAPQAGSQG     60
2           MFKRSVIAMACIFALSACGGGGGGSPDVKSADTLSKPAAPVVSEKETEAKEDAPQAGSQG     60
1           MFKRSVIAMACIFALSACGGGGGGSPDVKSADTLSKPAAPVVSEKETEAKEDAPQAGSQG     60
607         MFKRSVIAMACIFALSACGGGGGGSPDVKSADTLSKPAAPVVSEKETEAKEDAPQAGSQG     60
            ************.******************** ********:****:.***********

10          QGAPSAQGGQDMAAVSAENTGNGGAETADNPENKDEGTQNDMPQNAAESA----------     110
243         QGAPSTQGSQDMAAVSAENTGNGGAATTDKPKNEDEGPQNDMPQNA--------------     106
5           QGAPSAQGGQDMAAVSEENTGNGGAATADNPKNEDE-AQNDMPQNTAGTDSLTPNHTPAS     119
2           QGAPSAQGGQDMAAVSEENTGNGGAAATDKPKNEDEGAQNDMPQNAADTDSLTPNHTPAS     120
1           QGAPSAQGGQDMAAVSEENTGNGGAAATDKPKNEDEGAQNDMPQNAADTDSLTPNHTPAS     120
607         QGAPSAQGGQDMAAVSEENTGNGGAAATDKPKNEDEGAQNDMPQNAADTDSLTPNHTPAS     120
            *****:**.******* ******** ::*:*:*:**  *******:

10          -----------------------------------------------------NQTGNNQ     117
243         ---------------------------------------------GNTAAQGTNQAENNQ     121
5           NMPAGNMENQAPDAGESAQPENKPDMANAADGIQGDDPSADGENAGNTAAQGTNQAENNQ     179
2           NMPAGNMENQAPDAGESEQPANQPDMANTADGMQGDDPSAGGENAGNTAAQGTNQAENNQ     180
1           NMPAGNMENQAPDAGESEQPANQPDMANTADGMQGDDPSAGGENAGNTAAQGTNQAENNQ     180
607         NMPAGNMENQAPDAGESEQPANQPDMANTADGMQGDDPSAGGENAGNTAAQGTNQAENNQ     180
                                                                 **: ***

10          SAGSSDSAPASNPAPANGGGDFGRTNVGNSVVIDGPSQNITLTHCKGDSCDGDNLLDEEA     177
243         VGGSQNPAPSSNPNATN-GGNFGRVDLANGVLIDGPSQNITLTHCKSDSCNGDNLLSEEA     180
5           TAGSQNPASSTNPNATNGGGDFGRTNVGNSVVIDGPSQNITLTHCKGDPCNGDNLLDEEA     239
2           TAGSQNPASSTNPSATNSGGDFGRTNVGNSVVIDGPSQNITLTHCKGDSCSGNNFLDEEV     240
1           TAGSQNPASSTNPSATNSGGDFGRTNVGNSVVIDGPSQNITLTHCKGDSCSGNNFLDEEV     240
607         TAGSQNPASSTNPSATNSGGDFGRTNVGNSVVIDGPSQNITLTHCKGDSCSGNNFLDEEV     240
             .**.: * ::**  :* **:***.::.*.*:**************.* *.*:*:*.**.

10          PSKSEFDNLSESERMEKYKKDGK----SDKFTGFVADKLQMKGTNQYIIFYKPKTTSSAR     233
243         PSKSEFEQLSDEDKIKKYKKDG------EKFTGLVADRLQMKGTNQYIIFYKPKTTSSAR     234
5           PSKSEFEKLNESERIEKYKKDGK-----DKFVGLVATTVKMEGINKYIIFYTDKPPT---     291
2           QLKSEFEKLSDADKISNYKKDGKNDGKNDKFVGLVADSVQMKGINQYIIFYKPKPTSFAR     300
1           QLKSEFEKLSDADKISNYKKDGKNDGKNDKFVGLVADSVQMKGINQYIIFYKPKPTSFAR     300
607         QLKSEFEKLSDADKISNYKKDGKNDGKNDKFVGLVADSVQMKGINQYIIFYKPKPTSFAR     300
              ****::*.: :::.:*****      :**.*:**  ::*:* *:*****. *  :

10          FRRSARSRRSLPAEMPLIPVNQADTLIVDGEAVSLTGHSGNIFAPEGNYRYLTYGAEKLS     293
243         FRRSARSRRSLPAEMPLIPVNQADTLIVDGEAVSLTGHSGNIFAPEGNYRYLTYGAEKLP     294
5           --RSARSRRSLPAEMPLIPVNQADTLIVDGEAVSLTGHSGNIFAPEGNYRYLTYGAEKLS     349
2           FRRSARSRRSLPAEMPLIPVNQADTLIVDGEAVSLTGHSGNIFAPEGNYRYLTYGAEKLP     360
1           FRRSARSRRSLPAEMPLIPVNQADTLIVDGEAVSLTGHSGNIFAPEGNYRYLTYGAEKLS     360
607         FRRSARSRRSLPAEMPLIPVNQADTLIVDGEAVSLTGHSGNIFAPEGNYRYLTYGAEKLS     360
              *********************************************************

10          GGSYALRVQGEPAKGEMLAGTAVYNGEVLHFHTENGRPYPSRGRFAAKVDFGSKSVDGII     353
243         GGSYALRVQGEPSKGEMLAGTAVYNGEVLHFHTENGRPSPSRGRFAAKVDFGSKSVDGII     354
5           GGSYALSVQGEPAKGEMLAGTAVYNGEVLHFHTENGRPYPTRGRFAAKVDFGSKSVDGII     409
2           GGSYALRVQGEPSKGEMLAGTAVYNGEVLHFHTENGRPSPSRGRFAAKVDFGSKSVDGII     420
1           GGSYALSVQGEPAKGEMLAGTAVYNGEVLHFHTENGRPYPSRGRFAAKVDFGSKSVDGII     420
607         GGSYALSVQGEPAKGEMLAGTAVYNGEVLHFHTENGRSYPTKGRFAAKVDFGSKSVDGII     420
            ****** *****:************************  *::******************

10          DSGDDLHMGTQKFKAAIDGNGFKGTWTENGGGDVSGRFYGPAGEEVAGKYSYRPTDAEKG     413
243         DSGDGLHMGTQKFKAAIDGNGFKGTWTENGGGDVSGKFYGPAGEEVAGKYSYRPTDAEKG     414
5           DSGDDLHMGTQKFKAAIDGNGFKGTWTENGGGDVSGRFYGPVGEEVAGKYSYRPTDAEKG     469
2           DSGDGLHMGTQKFKAAIDGNGFKGTWTENGGGDVSGKFYGPAGEEVAGKYSYRPTDAEKG     480
1           DSGDDLHMGTQKFKAAIDGNGFKGTWTENGSGDVSGRFYGPAGEEVAGKYSYRPTDAEKG     480
607         DSGDDLHMGTQKFKAVIDGNGFKGTWTENGGGDVSGRFYGPAGEEVAGKYSHRPTDAEKG     480
            ****.**********.**************.*****:****.*********:********

10          GFGVFAGKKEQD      425
243         GFGVFAGKKEQD      426
5           GFGVFAGKKEQD      481
2           GFGVFAGKKEQD      492
1           GFGVFAGKKEQD      492
607         GFGVFAGKKEQD      492
            ************
```

Figure S2. Comparison of amino acid sequences of cross-reactive NHBA variants (full-length) with the sequence of NHBA peptide, ID 2, included in the 4CMenB vaccine. Polymorphic sites found in the C-terminal domain are indicated in green. Alignment of amino acid sequences constructed in Clustal Omega (https://www.kelleybioinfo.org/algorithms/tutorial/TAli2.pdf). An asterisk "*" indicates fully conserved amino acid position, two dots ":" indicate positions with conservation between amino acids with similar properties and one dot

"." indicates positions with conservation between amino acids with weakly similar properties. The number of last amino acid residue in line is indicated on the right.

```
NHBA, ID                          Sequence

197    ARFRRSARSRRSLPAEMPLIPVNQADTLIVDGEAVSLTGHSGNIFAPEGNYRYLTYGAEK    354
43     ARFRRSARSRRSLPAEMPLIPVNQADTLIIDGEAVSLTGHSGNIFAPEGNYRYLTYGAEK    353
47     ARVRRSARSRRSLPAEMPLIPVNQADTLIVDGEVVSLTGHSGNIFAPEGNYRYLTYGAEK    352
120    ARVRRSARSRRSLPAEMPLIPVNQADTLIVDGEAVILTEHPGNIFAPEGNYRYLTYGAEK    337
6      ARFRRSARSRRSLPAEMPLIPVNQADTLIVDGEAVSLTGHSGNIFAPEGNYRYLTYGAEK    292
9      ARFRRSARSRRSLPAEMPLIPVNQADTLIVDGEAVSLTGHSGNIFAPEGNYRYLTYGAEK    292
17     ARVRRSARSRRSLPAEMPLIPVNQADTLIVDGEAVSLTGHSGNIFAPEGNYRYLTYGAEK    291
18     ARVRRSARSRRSLPAEMPLIPVNQADTLIVDGEAVSLTGHSGNIFAPEGNYRYLTYGAEK    291
31     ---TRSARSRRSLPAEIPLIPVNQADTLIVDGEAVSLTGYSGNIFAPEGNYRYLTYGAEK    285
63     ---TRSARSRRSLPAEIPLIPVNQADTLIVDGEAVSLTGHSGNIFAPEGNYRYLTYGAEK    285
30     ----RSARSRRSLPAEMPLIPVNQVDTLIVDGEAVSLTGHSGNIFAPEGNYRYLTYGAEK    346
2      ARFRRSARSRRSLPAEMPLIPVNQADTLIVDGEAVSLTGHSGNIFAPEGNYRYLTYGAEK    358
112    -RSARSRRLRRSLPAEMPLIPVNQADTLIVDGEAVILTGHSGNIFAPEGNYRYLTYGAEK    351
           ** * *******:*******.****:***.* **  : ******************

197    LSGGSYALSVQGEPAKGEMLAGTAVYNGEVLHFHMENGRPSPSGGRFAAKVDFGSKSVDG    414
43     LSGGSYALRVQGEPAKGEMLAGTAVYNGEVLHFHMENGRPSPSGGRFAAKVDFGSKSVDG    413
47     LSGGSYALRVQGEPAKGEMLAGTAVYNGEVLHFHTENGRSYPTRGRFAAKVDFGSKSVDG    412
120    LSGGSYALRVQGEPAKGEMLAGTAVYNGEVLHFHTENGRSYPTKGRFAAKVDFGSKSVDG    397
6      LSGGSYALSVQGEPAKGEMLAGTAVYNGEVLHFHTENGRSYPTKGRFAAKVDFGSKSVDG    352
9      LSGGSYALSVQGKPAKGEMLAGTAVYNGEVLHFHTENGRSYPTRGRFAAKVDFGSKSVDG    352
17     LSGGSYALSVQGEPAKGEMLAGTAVYNGEVLHFHTENGRSYPTRGRFAAKVDFGSKSVDG    351
18     LSGGSYALSVQGEPAKGEMLAGTAVYNGEVLHFHTENGRSYPTKGRFAAKVDFGSKSVDG    351
31     LSGGSYALSVQGEPAKGEMLAGAAVYNGEVLHFHMENGRPYPSGGRFAAKVNFGSKSVDG    345
63     LSGGSYALRVQGEPAKGEMLAGTAVYNGEVLHFHMENGRPSPFRGRFAAKVDFGSKSVDG    345
30     LSGGSYALRVQGEPAKGEMLAGTAVYNGEVLHFHMENGRPSPFRGRFAAKVDFGSKSVDG    406
2      LPGGSYALRVQGEPSKGEMLAGTAVYNGEVLHFHTENGRPSPSRGRFAAKVDFGSKSVDG    418
112    LSGGSYALRVQGEPAKGEMLAGTAVYNGEVLHFHMENGRPSPFRGRFAAKVDFGSKSVDG    411
       * ****** ***:*:*******:*********** **** *  ******:********

197    IIDSGDDLHMGTQKFKAVIDGNGFKGTWTENGGGDVSGRFYGPAGEEVAGKYSYRPTDAE    474
43     IIDSGDDLHMGTQKFKAVIDGNGFKGTWTENGGGDVSGRFYGPAGEEVAGKYSYRPTDAE    473
47     IIDSGDDLHMGTQKFKAVIDGNGFKGNWTENGGGDVSGRFYGPAGEEVAGKYSYRPTDAE    472
120    IIDSGDDLHMGTQKFKAAINGNGFKGTWTENGGGDVSGKFYGPAGEEVAGKYSYRPTDAE    457
6      IIDSGDDLHMGKQKFKAAIDGNGFKGTWTENGGGDVSGRFYGPAGEEVAGKYSYRPTDAE    412
9      IIDSGDDLHMGTQKFKAAIDGNGFKGNWTENGGGDVSGRFYGPAGEEVAGKYSYRPTDAE    412
17     IIDSGDDLHMGTQKFKAAIDGNGFKGTWTENGGGDVSGRFYGPAGEEVAGKYSYRPTDAE    411
18     IIDSGDDLHMGKQKFKAAIDGNGFKGTWTENGGGDVSGRFYGPAGEEVAGKYSYRPTDAE    411
31     IIDSGDDLHMGTQKFKAAIDGNGFKGTWTENGGGDVSGRFYGPAGEEVAGKYSYRPTDAE    405
63     IIDSGDDLHMGTQKFKAVIDGNGFKGAWTENSGGDVSGRFYGPAGEEVAGKYSYRPTDAE    405
30     IIDSGDDLHMGKQKFKAAIDGNGFKGTWTENGGGDVSGRFYGPAGEEVAGKYSYRPTDAE    466
2      IIDSGDGLHMGTQKFKAAIDGNGFKGTWTENGGGDVSGKFYGPAGEEVAGKYSYRPTDAE    478
112    IIDSGDDLHMGTQKFKAAIDGNGFKGAWTENSGGDVSGRFYGPAGEEVAGKYSYRPTDAE    471
       ****** .****.*****.*:****** ****.******:*********************

197    KGGFGVFAGKKEQD    488
43     KGGFGVFAGKKEQD    487
47     KGGFGVFAGKKEQD    486
120    KGGFGVFAGKKEQD    471
6      KGGFGVFAGKKEQD    426
9      KGGFGVFAGKKEQD    426
17     KGGFGVFAGKKEQD    425
18     KGGFGVFAGKKEQD    425
31     KGGFGVFAGKKEQD    419
63     KGGFGVFAGKKEQD    419
30     KGGFGVFAGKKEQD    480
2      KGGFGVFAGKKEQD    492
112    KGGFGVFAGKKEQD    485
       **************
```

Figure S3. Comparison of the amino acid sequences of NHBA C-terminal domains from the "None" group with corresponding sequence of the NHBA peptide, ID 2, included in the 4CMenB vaccine. Red indicates the positions at which substitutions occur in the sequences of each NHBA of this group, and blue indicates the remaining polymorphic sites in the C-terminal domain. Alignment of amino acid sequences constructed in Clustal Omega (https://www.kelleybioinfo.org/algorithms/tutorial/TAli2.pdf). An asterisk "*" indicates fully conserved amino acid position, two dots ":" indicate positions with conservation between amino acids with similar properties and one dot

"." indicates positions with conservation between amino acids with weakly similar properties. The number of last amino acid residue in line is indicated on the right.

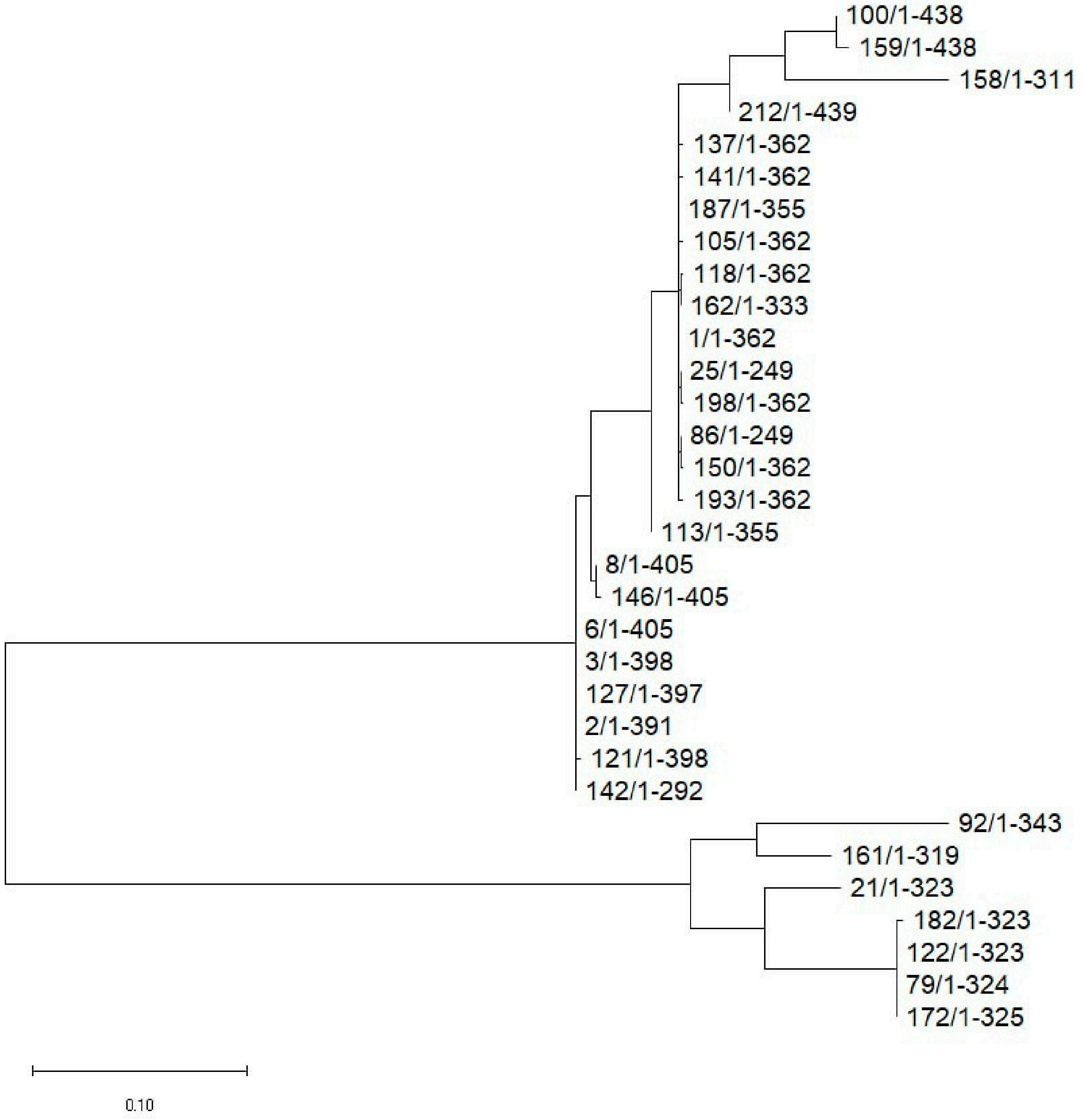

Figure S4. The tree of the NadA. Multiple alignments of amino acids sequences produced by the MUSCLE algorithm to construct phylogenetic trees of the NadA antigen. The trees were constructed using the Maximum Likelihood algorithm and visualized in MEGA software.
