# Supplementary figures and images for: Bioinformatics Analysis of Global Diversity in Meningococcal Vaccine Antigens over the Past 10 Years: Vaccine Efficacy Prognosis
Source: Med Sci (Basel). 2023 Dec 1;11(4):76. doi: 10.3390/medsci11040076 (PMC10744425; doi:10.3390/medsci11040076)

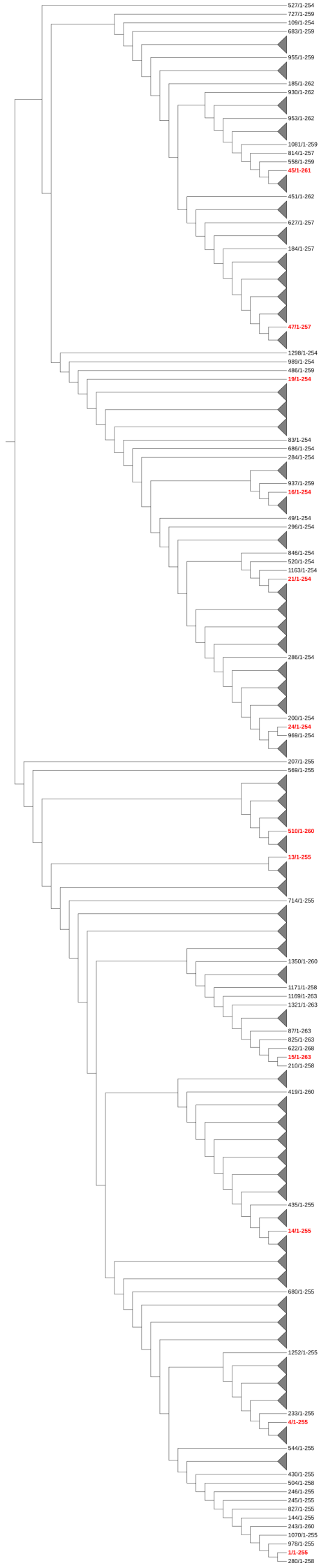

Supplement: Supplementary file 1 [file medsci-11-00076-s001.zip › File PDF S1 the global tree of fHbp alleles.pdf]

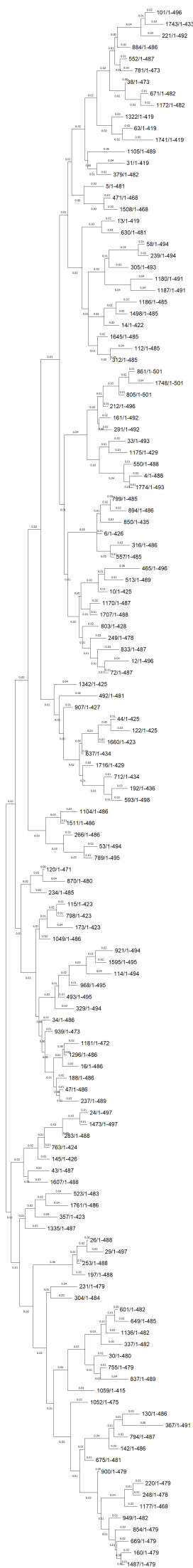

Supplement: Supplementary file 1 [file medsci-11-00076-s001.zip › File PDF S3 the tree of the NHBA.pdf]
