# Supplementary material for: Bioinformatics Analysis of Global Diversity in Meningococcal Vaccine Antigens over the Past 10 Years: Vaccine Efficacy Prognosis
Source: Med Sci (Basel). 2023 Dec 1;11(4):76. doi: 10.3390/medsci11040076 (PMC10744425; doi:10.3390/medsci11040076)

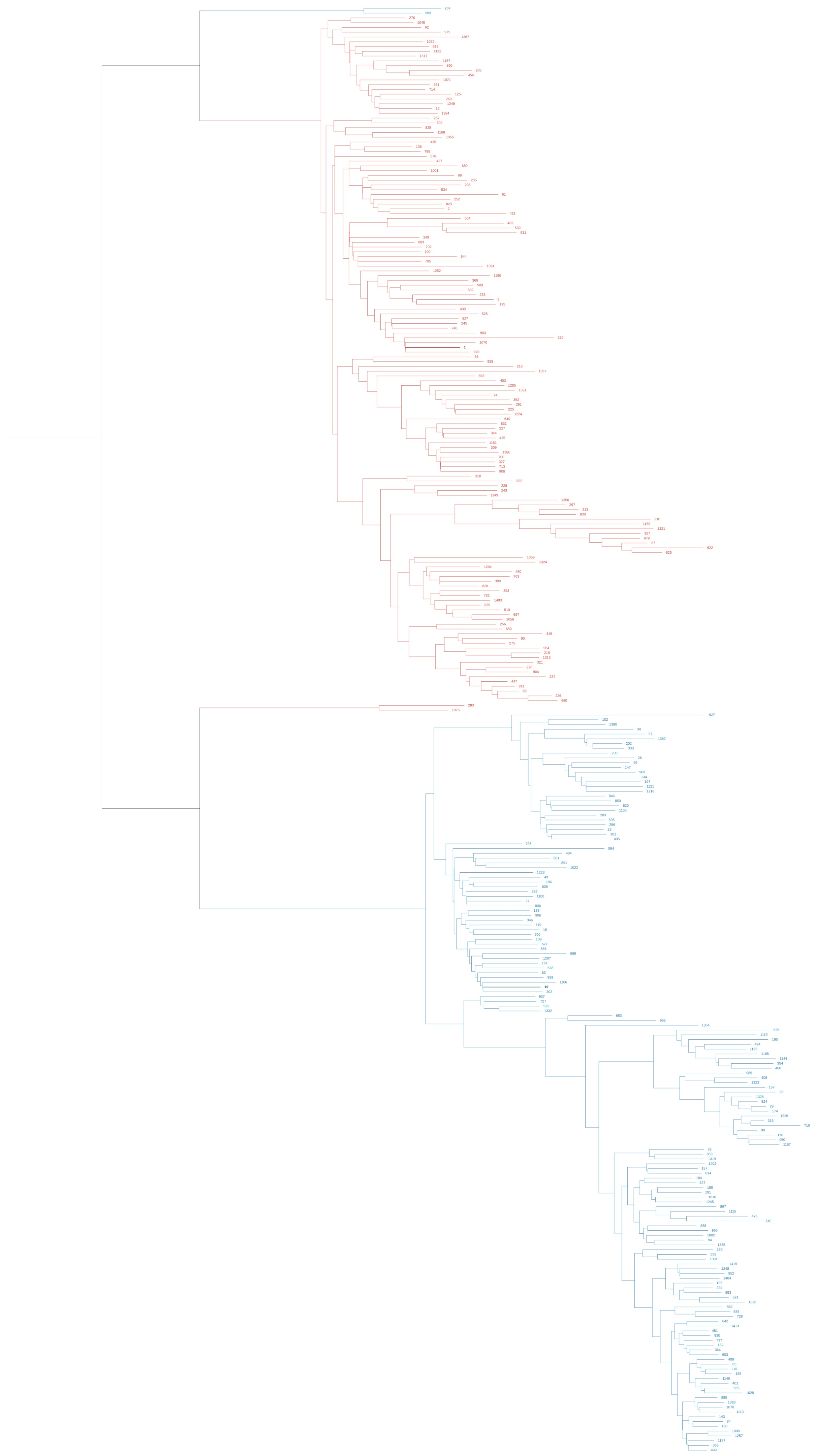

Supplement: Supplementary file 1 [file File PDF S2 the tree of the insufficient data_group of fHbp peptides .pdf › File PDF S2 the tree of the insufficient data_group of fHbp peptides .pdf]
